# An undiagnosed bilateral anterior shoulder dislocation after a seizure: a case report

**DOI:** 10.1186/1757-1626-1-342

**Published:** 2008-11-21

**Authors:** N Lasanianos, G Mouzopoulos

**Affiliations:** 1 Department of Trauma and Orthopedic Surgery, General Hospital of Athens "Evangelismos", 45-47 Ypsilantoy St., 10676, Athens, Greece

## Abstract

**Introduction:**

Late diagnoses of orthopaedic injuries after epileptic crisis are a matter of concern. The rarity of correlation between seizure and specific trauma incidences such as bilateral anterior shoulder dislocation, may lead to improper estimation of the patient's clinical state, wrong treatment and unpleasant complications.

**Case presentation:**

We report the rare case of an undiagnosed bilateral anterior shoulder dislocation in an epileptic young man of 25 years of age. The way of treatment is described as well as the treating alterations, if needed, because of the 3 weeks delay from injury. The article focuses on the reasons of the non-diagnosis at the first place and proposes a possible explanation for the mechanism of the injury. This is the second documented case of a missed bilateral anterior shoulder dislocation following a seizure and the first one that was treated not earlier than 3 weeks post injury.

**Conclusion:**

Although not a matter of routine, the high importance of radiographic control after seizure, in case of suspicion, is concluded. The etiology causing the injury shall not disorientate the doctors from the possible diagnoses.

## Background

Unilateral shoulder dislocation is the most common concerning 85% of all dislocations. Ninety-five per cent of all dislocations are anterior and 15% of them are combined with greater tuberosity fractures [1]. The most common bilateral shoulder dislocation is posterior resulting from seizure or convulsion due to epilepsy, electric shock or other reasons [2,3]. Simultaneous bilateral anterior shoulder dislocation is usually of traumatic origin and occurs rarely [4,5]. Seizure has been charged in the past for bilateral anterior shoulder fracture-dislocation [1,6,7]. Up to our knowledge though, this is the first case concerning treatment of such an undiagnosed post-seizure injury, three weeks after its occurrence. The difficulties of the diagnosis as well as the treatment are been described. A possible explanation for the mechanism of the injury is stated.

## Case presentation

**Figure 1:**
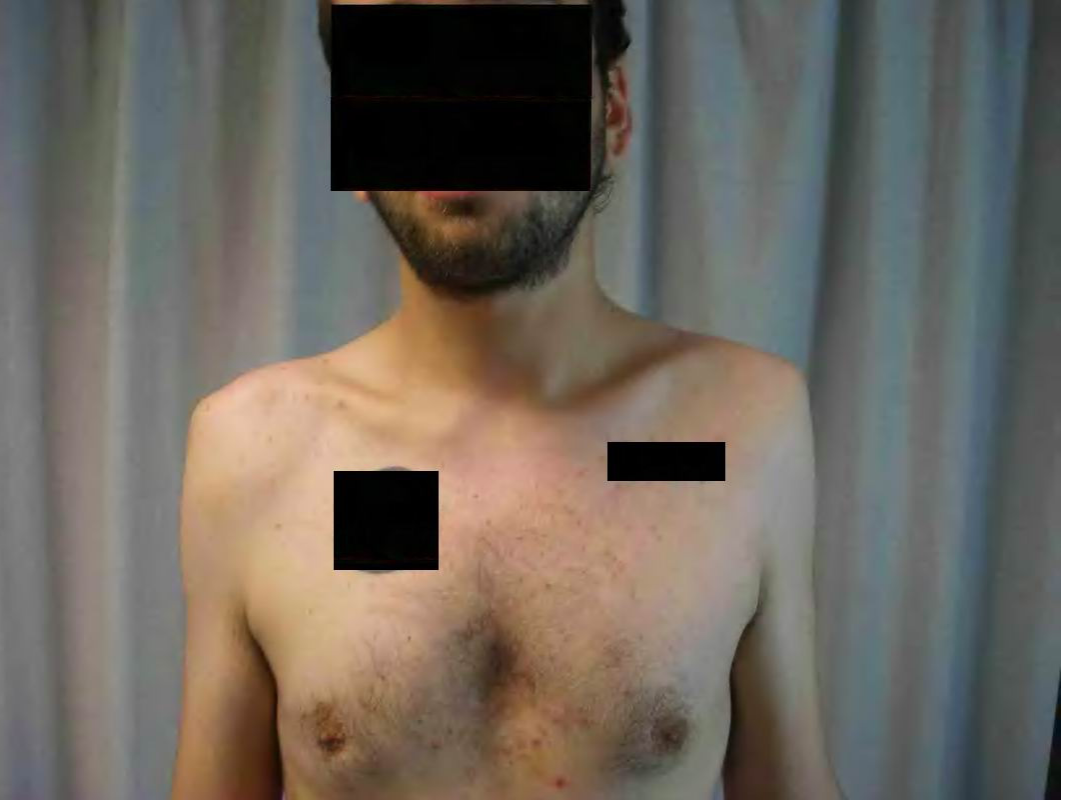
The clinical view of a 3 weeks neglected bilateral anterior shoulder dislocation after a seizure.

**Figure 2:**
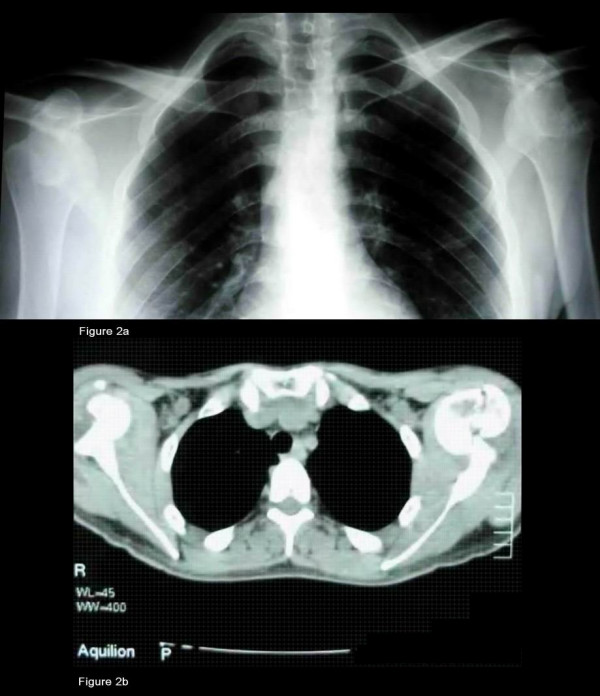
**A) Radiographic control before reduction.B) **CT scan pre reduction and ORIF (Open Reduction & Internal Fixation) showing a great tuberosity fracture of the left humerus and Hill Sachs lesions at both sites.

A 25 years old male patient presented for examination. Several incidents of Grand Mal epileptic seizure were referred with the last one three weeks ago. After the last episode both shoulders' range of motion was regressed and pain was elicited with movement. The patient visited a hospital at that point and without radiographic control performed, the diagnosis of bilateral shoulder contusions was posed. About three weeks later and as the patient realised that his injury was irreversible, he presented to the emergency room of our hospital. Clinical examination made clear that bilateral anterior shoulder dislocation was the point. (Fig. 1). No neurovascular injury was diagnosed in any arm. At the time of examination the patient was able to perform flexion and abduction of both arms up to 60 degrees as well as almost full external rotation with no pain restraint. From the patient's history no previous incidence of shoulder dislocation was referred. Radiographic control and Computed Tomography Scanning revealed the dislocations as well as a sizeable greater tuberosity fracture of the left humerus and Hill Sachs lesion at both sides. (Fig. 2a–2b).

**Figure 3:**
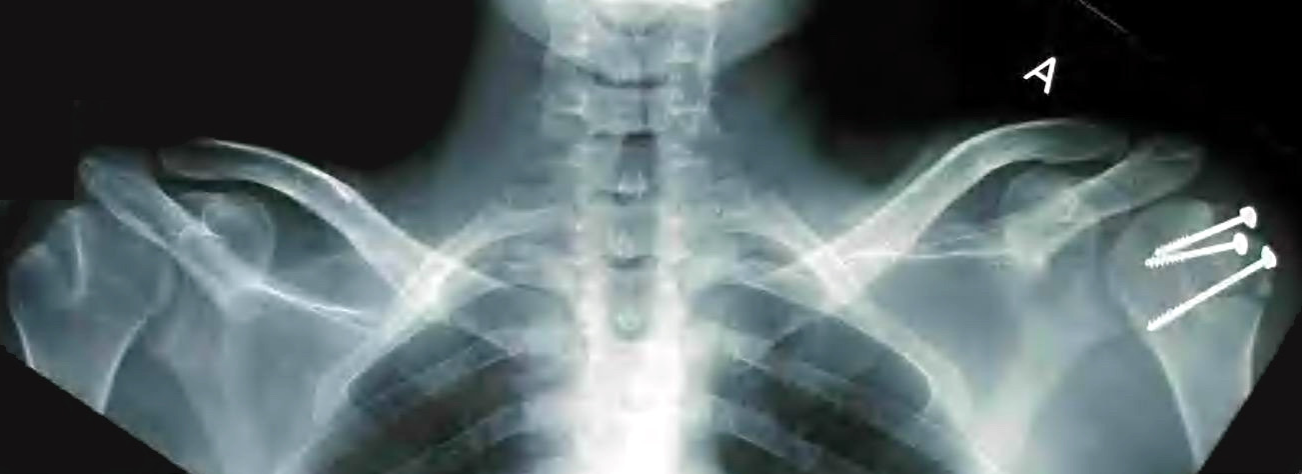
Post surgery radiographic control.

Bilateral reductions under general anaesthesia and internal fixation of the left greater tuberosity fracture were performed (Fig. 3). The reductions were easy to perform and shoulders' stabilisation was not carried out. No post manipulation neurovascular deficit was observed. Ultrasonography performed 4 days post surgery confirmed the integrity of rotator cuffs in both shoulders. Broad arm polyslings in abduction and internal rotation were used for 2 weeks and progressive mobilization started with pendulum exercises, forward flexion and abduction. A physiotherapy program of muscle enforcement was added at 3 weeks. The recovery was successful and after 2 months the patient had regained a normal range of motion in both shoulders with a minor lack at the last degrees of flexion and internal rotation of the left shoulder. Four months post-operatively the range of motion was fully recovered bilaterally and at the final follow-up, 2 years post surgery, the patient had not undergone any recurrent dislocation.

## Discussion

Anterior shoulder dislocations, unlike posterior, happen, almost always, secondary to trauma. Posterior shoulder dislocations usually occur following unbalanced muscle contractions (electric shock, epileptic seizure etc) [5,8]. The reason why the shoulder dislocates anteriorly after trauma is that as the arm extends and abducts, impingement of the greater tuberosity on the acromion levers the humeral head out of the glenoid [5]. Moreover the rotator cuff pushes downwards the humeral head which is finally displaced anteriorly by the flexors and external rotators. The posterior dislocations are more common after seizure since the contraction of the relatively weak external rotators and the posterior fibers of the deltoid are overcome by the more powerful internal rotator. The succeeding adduction and internal rotation usually causes the humeral head to dislocate posteriorly [8]. One suggestion about bilateral anterior dislocation following a seizure is that this may occur not during the muscle contractions but from the trauma of the shoulders striking the floor, after the collapse [8]. Bilateral occurrence of anterior shoulder dislocation is rare because almost always one extremity takes the brunt of the impact during the trauma incidence [5,8]. In our report we may suggest that loss of consciousness after the seizure did not allow the patient to react and reflexly protect one of his arms by exposing the other.

Associated fracture of the greater tuberosity occurs in 15% of the anterior dislocation cases and indicates an associated rotator cuff tear. If the greater tuberosity fracture is displaced the diagnosis of a rotator cuff tear is almost certain [9]. This may cause long term instability and functional impairment if the fragment is not anatomically reduced [5]. Thus internal fixation after the reduction must be the rule in such cases.

Closed reduction of a neglected anterior shoulder dislocation can be performed only up to six weeks post injury. After this period the danger of an iatrogenic fracture or neurovascular damage raises too high and operative procedures shall be followed. [10]

In our case the 3 weeks interval between the injury and the rehabilitation did not seem to influence the final functional outcome. Shoulder stabilization was not performed but the patient experienced no recurrent dislocation after two years of follow-up. As the shoulder is a muscle dependant joint one could suggest that when rotator cuff tears can be excluded, a proper physiotherapy program of muscle enforcement alone could be sufficient for a very good functional result, even in neglected cases like this one.

The literature suggests that over 10% of documented bilateral anterior shoulder dislocations following trauma were diagnosed late [5]. As there is a greater awareness of anterior shoulder dislocations for trauma patients, it would be reasonable to assume that there may be a higher incidence of delayed diagnosis of such an injury following a presentation with an indirect complaint, such as a seizure. The unusual presentation combined with the patient's post-ictal discomfort and drowsy state may potentially delay the diagnosis. As this could affect the prognosis, early recognition is vital. [8]

## Conclusion

Bilateral anterior fracture dislocation is a rare injury following seizure but emergency room doctors shall be familiar with this clinical situation. The etiology causing the injury shall not disorientate the doctor from the possible diagnoses. Although full musculoskeletal examinations are not routinely performed following a seizure, radiographic control of the shoulders shall be indispensably performed in case of suspicion.

## Consent

The authors of the study herein state that written informed consent was obtained from the patient for publication of this case report and any accompanying images. A copy of the written consent is available for review by the Editor-in-Chief of this journal.

## Competing interests

The authors declare that they have no competing interests.

## Authors' contributions

NL collected the data and drafted the manuscript. GM has been involved in revising the manuscript for important intellectual content. All authors read and approved the final manuscript.
